# Congenital Transmission of *Toxoplasma gondii* After Experimental Reinfection With Brazilian Typical Strains in Chronically Infected Sheep

**DOI:** 10.3389/fvets.2019.00093

**Published:** 2019-04-02

**Authors:** Daniela Pontes Chiebao, Hilda Fátima Pena, Danielle Passarelli, Thiago Santín, Lidia Hildebrand Pulz, Ricardo Francisco Strefezzi, Anaiá Paixão Sevá, Camila Marinelli Martins, Estela Gallucci Lopes, José Henrique Hildebrand Grisi Filho, Solange Maria Gennari, Rodrigo Martins Soares

**Affiliations:** ^1^Department of Preventive Veterinary Medicine, Faculty of Veterinary Medicine and Animal Science (FMVZ), University of São Paulo, São Paulo, Brazil; ^2^Department of Veterinary Medicine, Faculty of Animal Science and Food Engineering (FZEA), University of São Paulo, São Paulo, Brazil; ^3^Department of Animal Reproduction, Faculty of Veterinary Medicine and Animal Science (FMVZ), University of São Paulo, São Paulo, Brazil; ^4^Department of Pathology, Faculty of Veterinary Medicine and Animal Science (FMVZ), University of São Paulo, São Paulo, Brazil

**Keywords:** toxoplasmosis, oocysts, ovine, abortion, vertical transmission

## Abstract

*Toxoplasma gondii* is a cause of congenital diseases, miscarriages and stillbirths in production animals. In Brazil, non-archetypal genotypes of the parasite may be related to severe disease. Experimental infection with *T. gondii* was studied in sheep to analyse congenital transmission-related parameters in reinfections with different Brazilian parasite strains. Thirteen *T. gondii-*seronegative sheep were orally infected with 2 × 10^3^oocysts for the primary infection: G1 (4 animals) were inoculated with TgCatBr71 strain (Type BrI genotype) and G2 andG3 (5 and 4 animals, respectively) withTgCatBr60 strain (Type BrIII genotype). After chronification of infection, the animals were impregnated. A second infection was performed after 60 days of gestation. TheG1 andG3 animals were inoculated withTgCatBr60BrIII and the G2 animals withTgCatBr71 BrI oocysts. The effects of reinfection were compared with a control group (5 animals) through physical examination, ultrasound imaging and serology. Ovine experimental infections were evaluated using mouse bioassays, molecular analysis, serological tests, histopathology, and immunohistochemistry. No abortions occurred; a seropositive lamb and a mummified fetus from G2-BrIIIxBrI were produced. The vertical transmission rate detected in lambs from chronically infected sheep was 31.6% (6/19). It is demonstrated that reinfection and subsequent congenital transmission occured in one sheep with a primary Brl infection challenged with BrIII genotype of *T. gondii*. In a twin pregnancy from G2-BrIIIxBrI, congenital transmission from a latent infection was detected in both lambs. Congenital transmission could not be tracked in three lambs. Overall, previous *T. gondii* infection may fail to protect against congenital transmission from a reinfection and primary infection induced insufficient protection against vertical transmission which must be taken into account in decision-making for the use of seropositive animals as breeders. Similar trials with larger groups and contemplating host cellular immune response studies should be conducted to evaluate the actual impact of *T. gondii* reinfection involving different strains in sheep.

## Introduction

*Toxoplasma gondii* is a cosmopolitan protozoan of the Apicomplexa phylum and the aetioagent of toxoplasmosis. Its definitive hosts are felids (1), although hundreds of animal species, ranging from birds to mammals, including humans, may act as intermediate hosts (2). This parasite is transmitted through three main routes: (1) ingestion of oocysts eliminated in the feces of felids, which become infectious in the environment and contaminate water, soil, and food; (2) consumption of raw or undercooked meat of intermediate hosts containing viable tissue cysts with bradyzoites; and (3) congenital transmission via tachyzoites from the maternal host reaching the placenta and fetus (3).

Because *T. gondii* causes diseases with varying degrees of severity in its hosts, it is considered an important public and animal health problem and is recognized as the main parasitic cause of abortions in sheep (4). Toxoplasmosis in sheep usually clinically manifests after primary infection of a pregnant female and may lead to fetal death, mummification, abortion, or stillbirth, depending on the gestation stage (5). Although congenital transmission derived from primary infection is considered the most common route (6), transplacental transmission may also occur after recurrence of persistent infection with reactivation of latent cysts in the maternal organism (7).

Early studies with ewes naturally infected and experimentally infected with oocysts have indicated that sheep only experience reproductive problems after the first infection because, once infected, they produce an efficient and long-lasting cellular immune response that prevents the occurrence of congenital transmission or renders this event uncommon (5, 8). Studies based on the molecular diagnosis of *T. gondii*, without its isolation, have suggested that primary infection fails to provide protection against subsequent infections, resulting in a risk of vertical transmission in chronically infected animals (6, 9–11). In Brazil, studies have also indicated that experimental congenital transmission of *T. gondii* commonly occurs during various stages of gestation in chronically infected sheep (12), but these studies did not identify the strain genotype responsible for infection.

Different *T. gondii* variants may have different biological behaviors regarding the mode of transmission, virulence and ability to cause abortions in successive pregnancies in sheep (10, 13). *T. gondii* is a ubiquitous agent with high genotypic diversity and a peculiar population structure. The Type II and Type III clonal archetypes prevail in North American and European populations, whereas the number of prevalent genotypes is much higher in other parts of the world, particularly in South America (14, 15). An increased occurrence of the Types BrI, BrII, and BrIII non-archetypal genotypes was reported in sheep in Brazil (16, 17), although the archetypal Type II was recently described (18). The various *T. gondii* genotypes may also lead to different immune responses, as shown in a study in which lamb vaccination against toxoplasmosis, while drastically reduced parasite burden, failed to prevent reinfection when the animals were orally challenged with oocysts from a strain of another genotype (19).

Considering the questions regarding the occurrence of congenital toxoplasmosis transmission in sheep and the vast genotypic diversity of *T. gondii* in Brazil and South America, an experimental study is needed in sheep to analyse these parasite variables, to provide insights into the biological behavior of the variants prevalent in Brazil and to compare the results with data on the widely studied variants (archetypes I, II, and III). This study reports for the first time the outcomes of experimental oral infections of sheep with *T. gondii* oocysts from two field isolates presenting typical Brazilian genotypes, followed by reinfection of these animals with oocysts with a genotype different from that used in the primary infection. The sheep were impregnated between primary infection and reinfection to assess the effects of reinfection with a strain of a different genotype during pregnancy. Molecular diagnosis was performed to identify the strain of the parasite established after reinfection.

## Methods

### *T. gondii* Isolates

The TgCatBr71 (ToxoDB RFLP#6 or Type BrI) andTgCatBr60 (ToxoDB RFLP#8 or Type BrIII) isolates were selected for the experimental infection. Although the outcome of the disease may be different among species, these isolates were chosen because they show high genetic divergence and different pathogenicity in mice and occur at high frequencies in Brazil (17, 20–22). Both isolates were obtained by Pena et al. (20) and preserved in liquid nitrogen (−196°C).

*Toxoplasma gondii* tachyzoite and bradyzoite thawed saline suspensions were subcutaneously inoculated (1 mL per mice) into 50 female albino Swiss mice (25 for each isolate) to obtain chronically infected animals. Tachyzoites from the frozen tissues were not quantified whereas the purpose was reactivation in mice. When a second passage was necessary, the bradyzoite saline suspension contained 10 cists per inoculum. Mice inoculated with the Type BrI isolate were orally treated with sodium sulfadiazine (125 mg/100 mL) in the drinking water from day 0 post-inoculation (p.i.) to day 21 p.i.to induce the formation of tissue cysts, as Type BrI strains are normally virulent to mice (21).

The mice were kept in cages with five animals each and checked twice a day for signs of illness. Two months p.i., the mice were euthanized, and a small part of their brain cortex was microscopically examined for *T. gondii* cyst presence. Afterwards, 20 mice, 10 of each isolate, were selected for the cat bioassay.

### Bioassay in Cats

A litter of six cats descended from chipped breeders donated from a vaccine company (Laboratory Biovet) were used for the bioassay. After weaning, they tested seronegative for *T. gondii* (titer <16) and *Neospora caninum* (titer <50) according to the indirect immunofluorescence antibody test (IFAT). The cats were previously de-wormed, vaccinated and kept in individual cages with water *ad libitum* and commercial dry cat food according to the manufacturer instructions.

Euthanized mice chronically infected with *T. gondii* (2 months p.i.) were fed to cats aged 3–6 months old. Three cats were designated for each isolate. After 12 h of fasting, each cat ate at least two positive brains, and then, the whole mouse carcass was offered. Cats inoculated with different isolates were maintained in separate rooms and attended by different keepers four times a day for data and sample collections. Stool samples were examined daily for 30 days p.i.to detect oocysts using a sucrose flotation solution method (23). When identified, oocysts were recovered from the total feces, purified and concentrated for sporulation with 2% H_2_SO_4_ in an incubator at 25°C according to the method described by Dubey (2). The sporulated oocysts of each isolate were kept refrigerated at 4°C.Prior to use in the experiments, the oocysts were washed and resuspended in antibiotic (penicillin and streptomycin, 2000 IU and 200 μg/mL, respectively) and antifungal solutions. The suspension concentration was determined using a Neubauer chamber.

To confirm oocyst viability, 20 Swiss albino mice were divided into two groups of 10 animals each, and each group was orally inoculated with 50 oocysts per mouse with the TgCatBr71-Type BrI strain and the other with the TgCatBr60-Type BrIII strain. Animals had lung and liver samples collected to identify tachyzoites in slide imprints and brain material collected to identify tissue cysts in slide smears.

### Experimental Infection in Sheep

Eighteen Santa Inês ewe lambs ~1 year of age were purchased from a government breeding centre (Animal Science Institute of Nova Odessa/Sao Paulo—Instituto de Zootecnia de Nova Odessa, Sao Paulo State Department of Agriculture and Food Supply—Secretaria da Agricultura e Abastecimento do Estado de São Paulo) and quarantined at the experimental site on the USP campus in Pirassununga, São Paulo, Brazil. The animals were enclosed in roofed and fenced concrete pens, fed controlled diets and provided treated tap water. The lambs were immunized with anti-rabies and anti-clostridium vaccines and subjected to strategic parasite control.

Serum was collected for anti-*T. gondii*, anti-*N. caninum*, anti-*Brucella abortus*, anti-*B. ovis*, and anti-*Leptospira* spp. antibody detection analysis at the beginning and end of the quarantine. A reproductive evaluation (puberty diagnosis, oestrous phase, and uterus and ovary integrity, and size determinations) was performed using ultrasound (US) methods.

The animals were divided into four groups as follows: Group 1, four sheep (Nos. 1, 2, 3, and 4) received experimental primary infections with TgCatBr71-Type BrI oocysts and were then experimentally reinoculated with TgCatBr60-Type BrIII oocysts (G1-BrIxBrIII); Group 2, five sheep (Nos. 5, 6, 7, 8, and 9) received experimental primary infections with TgCatBr60-Type BrIII oocysts and were then experimentally reinoculated with TgCatBr71-Type BrI oocysts (G2-BrIIIxBrI); Group 3, four sheep (Nos. 10, 11, 12, and 13) received both experimental primary infection and reinoculation with TgCatBr60-Type BrIII oocysts (G3-BrIIIxBrIII); and Group 4, five sheep belonged to the uninfected control group (CG).

The G1, G2, and G3 sheep were inoculated with 2,000 oocysts per animal using an esophageal probe with the specific inoculum. The five CG animals were treated with physiological saline solution. All sheep were monitored once a day for 15 days p.i. and weekly afterwards to assess clinical parameters (rectal temperature, heart and respiratory rates, and lymph node status). Animals received proper treatment in the case of fever, pain, dehydration, and other common ovine affections, such as hoof disorders and caseous lymphadenitis. Serum was collected weekly until the second experimental infection was performed.

Four months after primary infection, all sheep were impregnated by natural mating using two *T. gondii-, N. caninum-, B. abortus-* ,and *B. ovis-*seronegative Santa Inês rams evaluated under quarantine as described above. Oestrus was synchronized using controlled internal drug release (CIDR®) intravaginal implants and estrogen and progesterone intramuscular applications (24). Pregnancy was confirmed by US 21 days after the mating period. In cases of oestrus detection, the same protocol was performed until the female became pregnant.

At 60 days of gestation, the G1, G2, and G3 sheep were subjected to a second experimental inoculation by esophageal probe with 2,000 *T. gondii* oocysts of the specific inoculum. The time of the second inoculation was chosen because the fetal heart rate can be measured by US at this age and this period has the highest incidence of abortions caused by *T. gondii* (25). The CG sheep were treated with physiological saline solution. The rectal temperature was assessed daily for 30 days p.i. and the other vital parameters as before. Serum collection and US (for fetal viability) were performed weekly for 15 weeks, until completion of the experiment.

Lambs were separated from their mothers after birth without ingesting the colostrum and were euthanized by jugular intravenous injection of veterinary euthanasia medication (T61®, MSD Animal Health, 4 mL/50 kg). The time point of the lamb sacrifice was chosen to minimize stress for the sheep and personnel, avoid unnecessary suffering and prevent stillborn losses. Adult sheep from all groups were euthanized after delivery by sanitary slaughtering at the school slaughterhouse on the USP campus in Pirassununga, São Paulo.

### Serological Tests

IFAT was performed for the anti-*T. gondii* and anti-*N. caninum* antibody analysis using 64 and 50 *T. gondii* antibody titres as the cut-off points for the adult sheep, respectively, and 16 as the cut-off point for the mice (26, 27). The *T. gondii* cut-off point used for the lambs was 4. Cell culture tachyzoites derived from the RH strain of *T. gondii* and the NC-1 strain of *N. caninum* were used as the antigens. Serodiagnosis for anti-*B. abortus* antibody detection in adult sheep was performed using the Rose Bengal-stained rapid plate seroagglutination with buffered acidified antigen (BAA) test (28). The agar gel immunodiffusion test using a commercial antigen was performed for *B. ovis* according to the manufacturer's instructions (Tecpar, Curitiba, Brazil). Adult sheep sera were also tested using the microscopic seroagglutination (MSA) micro-technique with live antigens (29) to test for antibodies against an antigenic battery of 22 *Leptospira* spp. serovars.

### Sheep Sample Collection After Infection

Prior to euthanasia, blood samples were collected from all animals to obtain serum. Following sheep and lamb euthanasia, the animals were inspected to identify any macroscopic alterations, and the tissues were collected aseptically. Sheep brain, heart, masseter, diaphragm, and semimembranosus and semitendinosus muscle samples were collected and pooled for the mouse bioassay. Parts of the brain, heart, liver, semimembranosus and semitendinosus muscle, placenta, and uterus samples were separated for the molecular and histopathological tests. The lamb brain, heart, and semimembranosus and semitendinosus muscle samples were separated and pooled for the mouse bioassay. Brain, heart, liver, and submandibular, popliteal and mesenteric lymph node samples were collected for the molecular and histopathological tests.

### Histopathology and Immunohistochemistry

The tissue fragments described before were fixed in 10% formalin buffered with phosphate buffer solution (0.05 M NaH_2_PO_4_, pH 7.2–7.4) for 24 h at a 1:10 ratio and then transferred into 70% ethanol. The tissues were dehydrated, diaphanized, impregnated, cut into 4-μm-thick sections and stained using the haematoxylin and eosin (HE) method (30). Lesions observed, comparing with negative tissue slides, were described. Special staining by periodic acid-Schiff (PAS) reaction was performed to help identify the bradyzoites.

The method adapted from Atmaca et al. (31) was used to perform immunohistochemistry (IHC). After antigen recovery from the slides (sodium citrate buffer, pH 6.0, 2 min under pressure), the slides were immersed in 3% H_2_O_2_ for 30 min and in 5% skim milk for 30 min. A rabbit anti-*Toxoplasma* polyclonal antibody (Abcam, Cambridge, USA) at a 1:200 dilution (Code AB15170) and a kit consisting of labeled streptavidin biotin reagents (Dako LSAB® 2 System-HRP, Dako Cytomation, Carpinteria, CA, USA) including a biotinylated anti-rabbit and anti-mouse immunoglobulins and streptavidin conjugated to horseradish peroxidase (Code K0609) were used according to the manufacturer's instructions to demonstrate *T. gondii* antigens in the slides. The histological sections on the slides were counterstained with Harris haematoxylin, followed by washes, dehydration, and diaphanization for mounting in synthetic resin on a cover slip. Mice tissues from the oocysts viability test were used as a positive control. Negative-control (N) slides were incubated using the same protocol with rabbit IgG and processed simultaneously to rule out non-specific interactions. The interpretation of positive results was based in immunohistochemically labeled antigen visualization.

### Mouse Bioassay

For *T. gondii* isolation, a protocol previously described was followed (32). Briefly, a pool of 50 g of tissues from each sheep and lamb was digested separately in pepsin acid solution, the suspensions were filtered, centrifuged and neutralized with 1.2% NaHCO_3_ (pH 8.3), and the pellets were resuspended in antibiotics. Each suspension was used as a subcutaneous inoculum in groups of four 60-day-old albino Swiss mice (1 mL/animal) per ovine. In the case of twin gestation, a pool of organs from both lambs (L1 and L2) was prepared for a group of four mice. The groups of mice were kept in separate cages and observed daily, and the procedures were performed as described above. The surviving animals were euthanized 60 days p.i. Mouse heart and brain samples were collected and stored at −20°C for molecular analysis, and plasma was collected from the thoracic cavity for anti-*T. gondii* antibody detection by IFAT.

### Molecular Diagnosis of *T. gondii*

DNA was extracted from the sheep and mouse tissues using a DNeasy Blood and Tissue kit (Qiagen® Inc., USA). The Tissue Spin and Blood Spin protocols were followed according to the manufacturer's recommendations. Internal transcribed spacer 1 (ITS-1) sequences of Toxoplasmatidae were identified by polymerase chain reaction (PCR-ITS1) as previously described (33–35). Positive (RH strain tachyzoite DNA) and negative (ultrapure water) controls were included in each reaction. The amplified products were resolved by 2% agarose gel electrophoresis and stained with ethidium bromide.

Nested polymerase chain reaction-restriction fragment length polymorphism (nested PCR-RFLP) directed at the *T. gondii* ß-tubulin (BTUB) gene was also performed to differentiate BrI from BrIII sequences (21, 36). Reference samples for different genotypes were used as positive controls (RH, PTG, CTG, Cougar, MAS, and TgCatBr5 strain tachyzoite DNA) and ultrapure water as a reaction negative control. The amplified and restriction-digested products were resolved by 2.5% agarose gel electrophoresis and stained with SYBR® Safe DNA Gel Stain (Invitrogen, USA).

### Statistical Analysis

The area under the mean antibody titer curve was calculated for each group, and the results were assessed by two-way analysis of variance (ANOVA) to compare humoral response data between groups. Tests were considered significant when *P* < 0.05 using Statistical Package for the Social Sciences (SPSS) software, version 16 (SPSS Inc., SPSS for Windows, Chicago, IL, USA).

## Results

### *T. gondii* Inoculum Viability

All cats remained healthy, and none was observed with diarrhea or fever during the bioassay. Approximately 2.0 × 10^6^sporulated oocysts were obtained from the TgCatBr71 Type BrI isolate, and 1.2 × 10^7^oocysts were obtained from the TgCatBr60 Type BrIII isolate. Nine of the ten mice inoculated with the BrI genotype oocysts showed signs of acute toxoplasmosis between 9 and 13 days p.i., with tachyzoites detected in the lungs. These mice either suddenly died (eight cases) of acute disease or were euthanized as soon as unambiguously clinical sign compatible to acute toxoplasmosis was noticed (1 case). The tenth mouse did not show any clinical sign and was euthanized 35 days p.i. It was positive only in the serological examination by IFAT. The 10 mice inoculated with BrIII genotype oocysts showed clinical signs of disease (ruffled fur, arched back, and dehydration). Tissue cysts were observed in all these mouse brains. Thus, oocysts of both isolates were viable for sheep inoculation.

### Clinical Signs in Sheep

**Figure 1:**
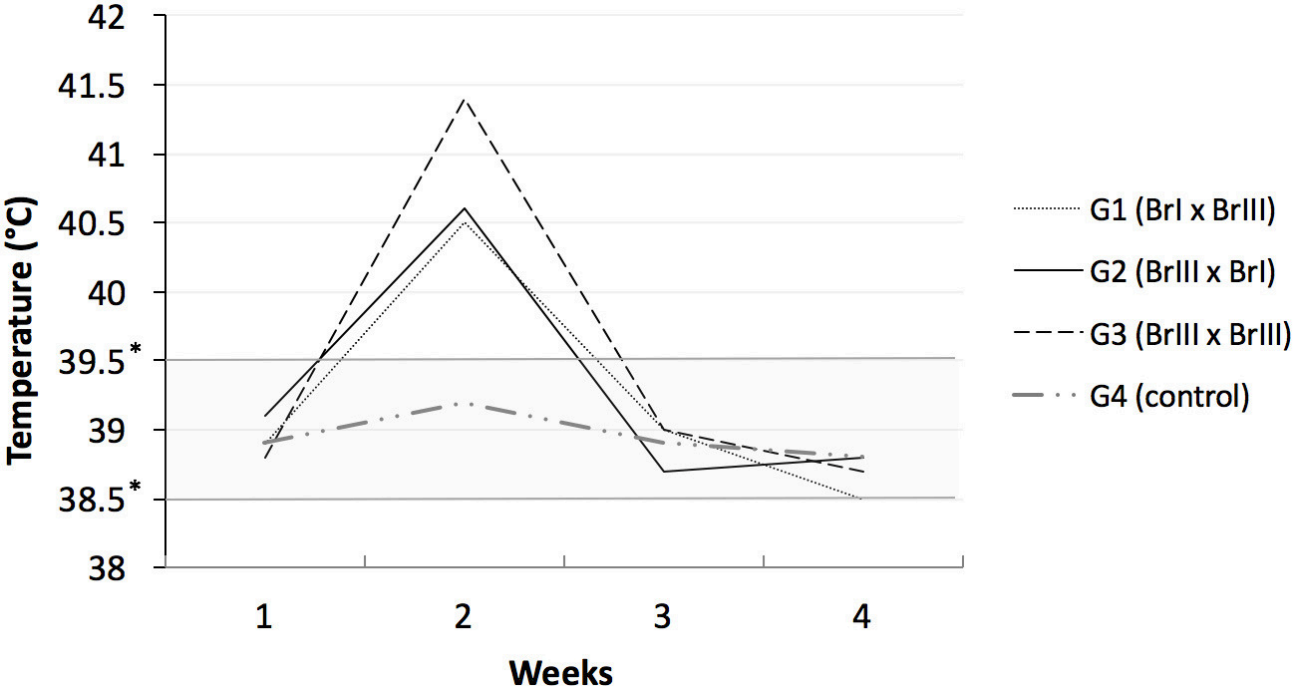
Sheep rectal temperatures after experimental prime infection using oocysts of *Toxoplamsa gondii* isolates TgCatBr71 (genotype BrI or TOXODB#6) and TgCatBr60 (genotype BrIII or TOXODB#8) ^*^low and high limits of normal temperatures.

Sheep from the experimental groups showed hyperthermia (Figure 1), hyporexia and apathy on days 7 to 9 after primary infection. No further changes in vital signs were detected throughout the remainder of the experiment. No abortions were observed during the study (Supplementary Material). The animals from all groups had full-term gestations according to the weekly US. A total of 24 lambs were born from the 18 impregnated sheep, including 19 from the 13 experimentally infected sheep. Sheep 5 (G2-BrIII x BrI) delivered a live lamb, and a 30-cm-long mummified fetus was expelled with the placental expulsion. Excluding the mummified fetus, all of the lambs were born healthy and without notable congenital deformities based on visual inspection. Ewes from G1, G2, and G3 produced 6, 8, and 5 lambs, respectively. A disseminated inflammatory reaction like fibrinoid deposit was observed in the placentas of all sheep from the three experimental groups, whereas the placentas of the CG animals showed no alterations.

In addition to the above findings, the presence of *T. gondii* DNA was detected in the placenta of sheep 11 (G3-BrIII x BrIII), and the BrIII genotype of the isolate was confirmed by PCR-RFLP. Pathology analysis by HE showed mononuclear infiltrate and antigen label by IHC was confirmed in the placentas of sheep 3 (G1-BrIxBrIII), sheep 6 (G2-BrIIIxBrI), and sheep 13 (G3-BrIIIxBrIII), although no signs of congenital transmission were observed in any of these animals.

### IgG Anti-*T. gondii* Antibody Serology

Two weeks after the primary infection, all 13 sheep from the experimental groups had anti-*T. gondii* antibody titres ≥64, with a peak up to 32,768 at 6 weeks p.i. and an antibody titer decline after 12 weeks p.i. These animals remained seropositive until the second infection. All CG sheep and offspring remained negative in the IFAT for *T. gondii* (titer <64). Lamb 2 of sheep 7 (G2-BrIII x BrI) was seropositive in the IFAT (titer = 64).

**Figure 2:**
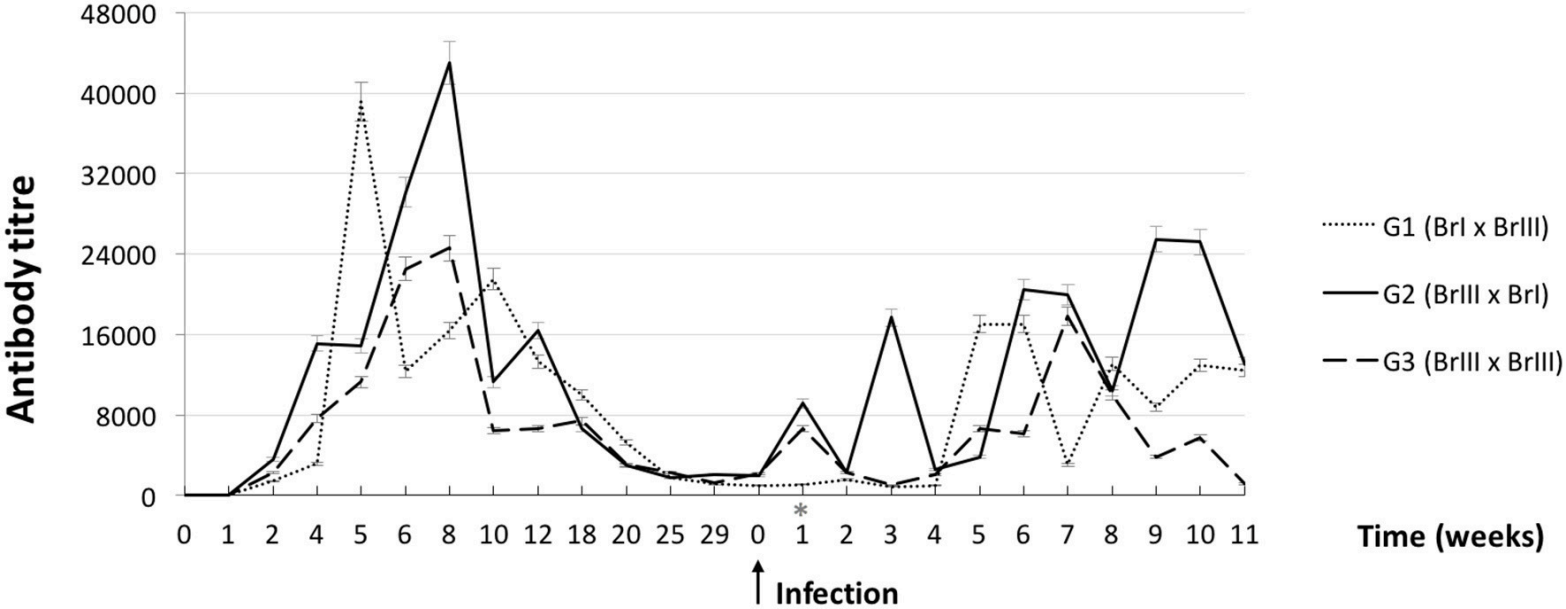
Curves of average anti-*Toxoplasma gondii* antibody titres from sheep after experimental prime infection using oocysts of the isolates TgCatBr71 (genotype BrI or TOXODB#6) and TgCatBr60 (genotype BrIII or TOXODB#8) followed by a second experimental infection during pregnancy (arrow) using oocysts from the same or from a different genotype, showing statistical significance (^*^) on group 1 (BrI x BrIII).

Figure 2 shows the mean humoural response kinetics in the experimental groups throughout the experiment. No intergroup differences in the mean antibody titres were detected in the first or second infection. However, when the intragroup mean titer variation throughout the experiment was tested, a difference between the first and second infections (*P* = 0.016) was observed in G1-BrI x BrIII, with mean titres significantly lower in the second infection compared with the first infection.

### Necropsy Data

Generalized lymphadenomegaly was observed in lamb 1 of sheep 1 (G1-BrIxBrIII), both lambs of sheep 7 and lamb 1 of sheep 8 (G2-BrIIIxBrI). One lamb of sheep 7 also had splenomegaly. The only offspring of sheep 10 (G3-BrIIIxBrIII) had enlarged popliteal lymph nodes and hepatomegaly. Hepatomegaly was the only finding in lamb 2 of sheep 11 (G3-BrIIIxBrIII).

### Sheep Infection With *T. gondii*

Brain samples from all 13 experimentally infected sheep were positive by PCR-ITS1. *T. gondii* was isolated from 10 animals; the exceptions were sheep 6, 8, and 9. All mice inoculated with tissues from the 13 sheep were IFAT positive (Table 1). Acute toxoplasmosis occurred in mice inoculated with tissues from sheep 2, 3, and 4 (G1-BrI x BrIII) (7/12) and from sheep 13 (G3-BrIII x BrIII) (1/4). No mouse inoculated with tissues from sheep 1 (G1-BrIxBrIII) became sick or died, but tissue cysts were observed in their brains. In all sheep in which different isolates were used (G1 and G2), the strain genotype obtained in the mouse bioassays corresponded to the isolate used in the primary infection except for sheep 1 (G1-BrI x BrIII) (Table 1) which is evidence of reinfection. In sheep 6, 8, and 9 (G2-BrIIIxBrI), genotyping was performed using DNA directly obtained from the sheep brain by PCR-RFLP. Genotyping was only successful for sheep 9, in which the Type BrIII genotype was obtained.

**Table 1:** Analysis of tissues from sheep experimentally infected with oocysts of TgCatBr71 (Type BrI genotype) and TgCatBr60 (Type BrIII genotype) *Toxoplasma gondii* isolates after a second infection following the chronification of a primary infection.

**Group**	**Sheep number**	**PCR-ITS1 from primary sample (brain)**	**Isolation from mouse bioassays**		**Isolates genotype (PCR-RFLP)/Mouse serology (IFAT titer>64)**
			**Tachyzoites (lungs) 16–26 days p.i**.	**Cysts (brain) 60 days p.i**.	
G1-BrIxBrIII	1	Positive	N	Positive	BrIII/Positive
	2	Positive	Positive	N	BrI/Positive
	3	Positive	Positive	N	BrI/Positive
	4	Positive	Positive	N	BrI/Positive
G2-BrIIIxBrI	5	Positive	N	Positive	BrIII/Positive
	6	Positive	N	N	ND/Positive
	7	Positive	N	Positive	BrIII/Positive
	8	Positive	N	N	ND/Positive
	9	Positive	N	N	BrIII/Positive
G3-BrIIIxBrIII	10	Positive	N	Positive	BrIII/Positive
	11	Positive^*^	N	Positive	BrIII/Positive
	12	Positive	N	Positive	BrIII/Positive
	13	Positive^**^	Positive	Positive	BrIII/Positive

ND, not done;

* also placenta;

** *also heart; N, negative*.

### Lamb Infections With *T. gondii*

**Figure 3:**
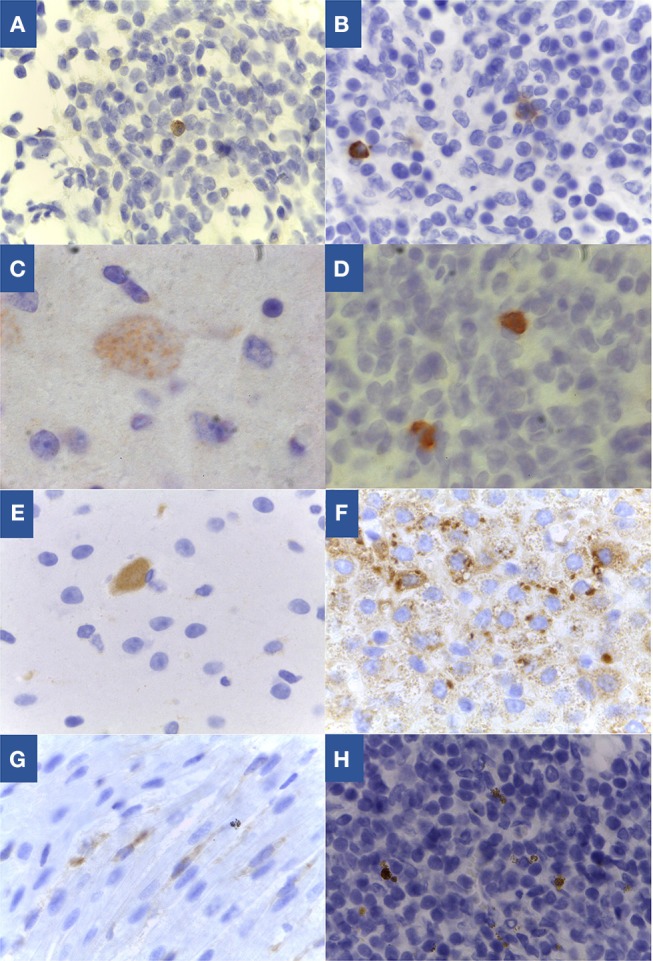
*Toxoplasma gondii* specific labeling by immunohistochemistry in tissues of congenitally infected lambs. **(A)** Lymph node from lamb 1 of sheep 1 from G1, 100x; **(B)** Lymph node from lamb 1 of sheep 7 from G2, 100x; **(C)** Brain from lamb of sheep 9 from G2, 100x; **(D)** Lymph node from lamb of sheep 9 from G2, 100x; **(E)** Brain from lamb of sheep 10 from G3, 100x; **(F)** Lymph node from lamb of sheep 10 from G3, 40x; **(G)** Heart from lamb of sheep 10 from G3, 100x; **(H)** Lymph node from lamb 1 of sheep 11 from G3, 100x.

Vertical transmission was confirmed in six lambs of the 19 offspring obtained (31.6%). Vertical transmission was defined if *T. gondii* was detect in fetuses by HE/IHC and/or PCR-RFLP and/or mouse isolation. Considering the total number of pregnancies, congenital transmission occurred in five of 13 (35.5%). Infection with *T. gondii* was confirmed in lambs born of sheep 1 (G1-BrI x BrIII); 7 (L1 and L2) and 9 (G2-BrIII x BrI); and 10 and 11 (G3-BrIII x BrIII). The rate transmission in G1 was the lowest, 16.7% (1/6), followed by G2 with 37.5% (3/8) and the highest in G3 in which two out of five lambs were congenitally infected (40%). Results from the analysis of tissues of confirmed *T. gondii*-infected lambs are outlined in Table 2 and Figure 3. For sheep 1 (G1-BrI x BrIII), the strain genotype involved in the infection of L1 was BrIII, indicating the occurrence of transplacental transmission from a second oocyst infection. Histopathology analysis revealed small loci of mononuclear inflammatory infiltrate in the liver parenchyma of lamb 1. For sheep 7 (G2-BrIII x BrI), genotyping showed that congenital transmission for L1 and L2 was caused by the TgCatBr60-Type BrIII strain and was therefore a reactivation of a latent BrIII infection. A few loci of necrosis and mononuclear inflammatory infiltrate was found in the liver of lamb 1 from sheep 7 (G2). Congestive lesions were detected in the heart from lamb of sheep 10 from G3.

**Table 2:** Results from analysis of tissues of *Toxoplasma gondii* infected lambs born by sheep with chronic toxoplasmosis after experimental infection using oocysts of TgCatBr71 (Type BrI genotype) and TgCatBr60 (Type BrIII genotype) isolates during pregnancy.

**Sheep group**	**Lamb (L)^#^**	IFAT^*^	**HE/IHQ (tissue)**	**PCR-RFLP genotype (tissue)**	**Isolation from mouse bioassays**		
					**Tachyzoites/cysts**	**PCR-RFLP genotype**	IFAT^**^
G1- BrIxBrIII	L1, Sheep 1	N	Positive (lymph node)	BrIII (lymph node)	N	ND	N
G2-BrIIIxBrI	L1, Sheep 7	N	Positive (lymph node)	BrIII (lymph node)	Positive	BrIII	Positive
	L2, Sheep 7	Positive	Positive (lymph node)	BrIII (lymph node, heart)	Positive	BrIII	Positive
	L, Sheep 9	N	Positive (brain, lymph node)	NS	N	NS	N
G3-BrIIIxBrIII	L, Sheep 10	N	Positive (brain, heart, lymph node)	NS	N	NS	N
	L1, Sheep 11	N	Positive (lymph node)	BrIII (lymph node)	N	NS	N

# L1 and L2 refer to twins; ND, not done;

* cut-off = 1:4;

** *cut off = 1:64; N, negative; NS, not successful*.

Analysis of the mummified fetus tissues were tried by PCR and HE/IHC but resulted negative probably due to sample decomposition.

## Discussion

Outbreaks of toxoplasmosis in sheep flocks may lead to extensive economic losses in both Brazil [e.g., in a farm in the state of Rio Grande do Sul wherein abortions occurred in 58.3% of the sheep (37)] and other countries [e.g., in the USA wherein a farm in the state of Texas experienced 78% reproductive losses due to abortions, mummified fetuses or peripartum deaths in 2005 (38)]. Determining the origin of congenital transmission (either from oocyst or from a latent infection) in these reports of outbreaks in farms was not possible. In our study investigating chronically infected sheep, the occurrence of the transplacental transmission derived from a primary infection was only shown in one animal of three infected offspring that could have the genotype assessed. The total percentage of vertical transmission in this study was lower than the percentage observed in outbreaks however, in general, the *T. gondii* infection status of sheep (primary infection or chronic infection) is unknown.

Compared with other vertical transmission studies based on diagnosis by PCR, which indicated rates higher than 60% in sheep without a defined history of toxoplasmosis (9, 39) or up to 48% in families of Charollais sheep with natural chronic infections (40), the results reported herein showed a lower rate of transmission that did not result in abortions or neonatal deformities, with the exception of one mummified fetus. Because these outcomes are the main characteristics related to economic losses resulting from disease in sheep flocks, the present study suggests that congenital transmission in chronically infected animals has no greater impact than transmission by ingestion of *T. gondii* oocysts from a contaminated environment, which was previously established (7, 41) as a source of infection for sheep that adversely affects the reproductive performance of sheep flocks. As 68% of the lambs (13/19) produced in this study were not infected, we concluded that the primary infection induces moderate protection against vertical transmission. In fact, this transmission route is considered non-negligible (7, 41) and should be considered in toxoplasmosis control programmes. It was stablished that two out of three infected lambs experienced transmission derived from reactivation of a latent infection what must have resulted from reactivation of the primary infection. Although the lambs were born clinically normal and almost all lacked anti-*T. gondii* antibodies, some tissue alterations were observed, primarily in the lymph nodes and liver (i.e., organs related to the immune response); these findings were corroborated by positive histological and PCR findings. Therefore, these animals could help maintain the life cycle of the parasite and possibly threaten human health. Additionally, as observed in humans (42–45), disease exacerbation could occur during lamb growth, and clinical signs might appear. In the present experiment no sheep had aborted after a second infection, although in six fetuses (from five sheep) we had collected evidence of *T. gondii* transmission. To know why the six vertical transmissions detected in this study did not result in miscarriages, it would be necessary to evaluate the cellular immune response of the sheep throughout the experiment, comparing with experimentally groups of sheep infected only once.

The gestation period in which the infection occurs may contribute to different results. In our experiment, we estimated that at the time of reinfection the sheep were at a stage where vertical transmission would have a high chance of causing abortion. A study using laboratory reference strains (ME49 and VEG) for experimental infection and reinfection at various gestation stages of Santa Inês sheep reported a high occurrence of congenital transmission and clinical signs in fetuses (12). Evaluating transmission at different time points (40, 80, and 120 days), no abortions were reported in the study, and only one macerated fetus (from the group reinfected at 120 days of gestation) and one mummified fetus (from the group reinfected at 40 days of gestation) were found of the 25 lambs obtained. These data are similar to the findings reported herein. In addition to the gestational time, the higher vertical transmission rate and congenital alterations may be related to the use of parasites with several laboratory passages, which may lead to increased agent virulence.

The higher pathogenicity of the Brazilian strains used in the present study might explain the reinfection on sheep 1 from G1 and further transmission of the second inoculum to the fetus. A study on congenital toxoplasmosis in pregnant rodents of the *Calomys* genus chronically infected and reinfected with BrI and BrII *T. gondii* strain genotypes showed that the immune response acquired during primary infection was insufficient to prevent vertical transmission of the challenging strain (46). As observed in the present study, congenital transmission from a latent infection occurred after challenge with the BrI genotype on G2. The pathogenicity of *T. gondii* infection appears to be dependent on the up and down-regulation of several genes involved in host immune systems (47–49) and this intricate process of regulating gene expression can be altered during reinfection so that future studies are welcome to investigate whether the BrI genotype parasite (pathogenic in mice) might modulate the host immune response during reinfection, thereby enabling the congenital transmission of the BrIII *T. gondii* strain genotype. In addition, this response may vary according to the host. For example, intestinal immunity might suffice to limit infection with the parasite in pigs vaccinated with *T. gondii* subunits (50). In humans, the parasite genotype may affect the occurrence of congenital toxoplasmosis (51). A cohort study in children from Brazil and Europe showed that toxoplasmic retinochoroiditis is more severe in Brazil, probably owing to the more virulent strains of the parasite (52).

The present study showed that primary infection with the BrI strain genotype might be insufficient to establish a long-lasting immune response because G1-BrIxBrIII showed significant differences in the antibody titres produced by sheep upon reinfection without the expected boosting (53), which might have contributed to sheep reinfection with a parasite of a different genotype.

Histopathological findings in new-born lamb tissues were observed at a lower intensity and in lower numbers than reports from previous studies with primary infections in pregnant ewes (54) and animals vaccinated and challenged with a different *T. gondii* genotype (19). This finding held true even with the high inoculum dose used in this study, with the exception of the hepatic alterations described in one G2-BrIII x BrI lamb. We hypothesized that primary infection with non-archetypal *T. gondii* parasites might be more effective in reducing clinical symptoms because challenged sheep from the experimental groups showed no abortions. In mice chronically infected with a genotype BrIII strain, it was observed diminished parasite spread and mortality after oral challenge with *T. gondii* cysts from non-archetypal virulent strains (55). Vertical transmission was lowest in G1 where only one lamb out of 6 was positive which is interesting for future vaccine study since the ewes received primary infections with oocysts of genotype BrI, and this variant is phylogenetically and biologically (based on the pathogenicity in mice) close to the type I archetype of the strain used in the commercial vaccine against toxoplasmosis in sheep.

## Conclusions

The evidence shows that previous *T. gondii* infection may fail to protect against congenital transmission from a reinfection. Although the congenital infection did not appear to have caused any reproductive loss and were not able to kill the fetuses, the *in utero* infected lambs were not followed, and the productive and reproductive prognosis of these animals was unknown. Overall, primary infection induced insufficient protection against vertical transmission which must be taken into account in decision-making for the use of seropositive animals as breeders. Similar trials with larger groups and contemplating host cellular immune response studies should be conducted to evaluate the actual impact of *T. gondii* reinfection on production animals.

## Data Availability

All datasets generated for this study are included in the manuscript and/or the Supplementary Files.

## Ethics Statement

All procedures performed in studies involving animals were in accordance with the Ethical Principles in Animal Research adopted by the College of Animal Experimentation (COBEA) and were approved (protocol number 2351/2011) by the Ethical Committee for Animal Welfare (CEUA), USP, São Paulo.

## Author Contributions

DPC, SMG, and RMS conceived and designed the experiment. DPC conducted the experiment. EGL completed the mice bioassay. DP completed the serology. TS completed the sheep clinical and reproductive examinations. HFJP completed the molecular analysis. LHP and RFS completed the histopathological analysis. JHHG, APS, and CMM completed the statisticalanalysis. DPC, HFJP, RFS, APS, CMM, JHHG, and RMS contributed to the interpretation of data. DPC and RMS wrote the paper. We confirm that the manuscript has been read and approved by all named authors and that there are no other persons who satisfied the criteria for authorship but are not listed. We further confirm that all of us have approved the order of authors listed in the manuscript.

### Conflict of Interest Statement

The authors declare that the research was conducted in the absence of any commercial or financial relationships that could be construed as a potential conflict of interest.
