# Supplementary material for: Congenital Transmission of Toxoplasma gondii After Experimental Reinfection With Brazilian Typical Strains in Chronically Infected Sheep
Source: Front Vet Sci. 2019 Apr 2;6:93. doi: 10.3389/fvets.2019.00093 (PMC6454189; doi:10.3389/fvets.2019.00093)
Supplement: Supplementary file 1 [file Table_1.docx]

Supplementary 1. Analysis of the offspring born from sheep with chronic toxoplasmosis and experimentally reinfected with typical Brazilian isolates of *Toxoplasma gondii* during pregnancy.

|  | Group | | | | | | | | | | | | | | |
| --- | --- | --- | --- | --- | --- | --- | --- | --- | --- | --- | --- | --- | --- | --- | --- |
|  | **G1-BrIxBrIII** | | | |  | **G2-BrIIIxBrI** | | | | |  | **G3-BrIIIxBrIII** | | | |
| Sheep identification | 1 | 2 | 3 | 4 |  | 5 | 6 | 7 | 8 | 9 |  | 10 | 11 | 12 | 13 |
| Abortions | 0 | 0 | 0 | 0 |  | 0 | 0 | 0 | 0 | 0 |  | 0 | 0 | 0 | 0 |
| Born lambs | 2 | 1 | 2 | 1 |  | 1 | 2 | 2 | 2 | 1 |  | 1 | 2 | 1 | 1 |
| Congenital defects | 0 | 0 | 0 | 0 |  | 0 | 0 | 0 | 0 | 0 |  | 0 | 0 | 0 | 0 |
| Mummified foetus | 0 | 0 | 0 | 0 |  | 1 | 0 | 0 | 0 | 0 |  | 0 | 0 | 0 | 0 |
| Lambs serology titre (IFAT) | N/N | N | N/N | N |  | N | N/N | N/64 | N/N | N |  | N | N/N | N | N |
| Mice per bioassay | 4 | 4 | 4 | 4 |  | 4 | 4 | 4 | 4 | 4 |  | 4 | 4 | 4 | 4 |
| 1. IFAT positive^a^ | 0 | 0 | 0 | 0 |  | 4 | 0 | 3 | 0 | 0 |  | 0 | 0 | 0 | 0 |
| 1. PCR positive | 0 | 0 | 0 | 0 |  | 0 | 0 | 1 | 0 | 0 |  | 0 | 0 | 0 | 0 |

N=negative (<4); ^a^cutt-off=64
